# Prodrug Integrated Envelope on Probiotics to Enhance Target Therapy for Ulcerative Colitis

**DOI:** 10.1002/advs.202205422

**Published:** 2022-12-11

**Authors:** Kun Zhang, Li Zhu, Yuan Zhong, Lixin Xu, Chunhui Lang, Jian Chen, Fei Yan, Jiawei Li, Juhui Qiu, Yidan Chen, Da Sun, Guixue Wang, Kai Qu, Xian Qin, Wei Wu

**Affiliations:** ^1^ Key Laboratory for Biorheological Science and Technology of Ministry of Education State and Local Joint Engineering Laboratory for Vascular Implants Bioengineering College of Chongqing University Chongqing 400030 P. R. China; ^2^ Chongqing University Three Gorges Hospital Chongqing Municipality Clinical Research Center for Geriatric diseases Chongqing 404000 P. R. China; ^3^ Institute of Life Sciences and Biomedical Collaborative Innovation Center of Zhejiang Province Wenzhou University Wenzhou Zhejiang 325035 P. R. China; ^4^ Jin Feng Laboratory Chongqing 401329 P. R. China

**Keywords:** lactobacillus rhamnosus GG (LGG), oral delivery, prodrug, surface decoration, ulcerative colitis

## Abstract

Ulcerative colitis (UC), affecting millions of patients worldwide, is associated with disorders of the gut microbiota. Probiotics‐based therapy positively regulating the community structure of gut microbiota is regarded as an efficient intervention for UC. However, oral probiotics delivery is restricted by limited bioactivity, short retention time, complex pathological condition, and single therapeutic efficacy. Here, a bioengineered probiotic decorated with a multifunctional prodrug coating is constructed to ameliorate the aforementioned shortcomings. The results of UC mice induced by dextran sulfate sodium demonstrate that the intrinsic features of the fabricated coating integrate gut microbes protection, colon‐targeted drug release, prolonged drug retention, and inflammation regulation. In parallel, the probiotics *Lactobacillus rhamnosus* GG (LGG) could regulate the composition of the gut microbiota and improve epithelial barrier function, thereby synergistically ameliorating UC. These results provide ample shreds of evidence of the therapeutic effect on UC, therefore, demonstrate a great promise as the potential therapeutic strategy for UC treatment.

## Graphical abstract

For improving the synergistical therapy in ulcerative colitis, the prodrug integrated envelope on probiotics is exploited to greatly protect the bacterial biological activity from gastrointestinal delivery, significantly enhance the target cargo release to reduce the local inflammation for repairing the intestinal barrier functions, and efficiently promote probiotics colonization to positively regulate the gut microbiota.

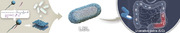

## 1 Introduction

The human intestinal tract is home to trillions of microbes that form a complex community referred to as the gut microbiota.^[^ ^1^, ^2^, ^3^ ^]^ Increasingly, researches have revealed that some gut microbes play vital roles in immunity modulation, homeostasis maintenance, and host health.^[^ ^4^, ^5^, ^6^, ^7^ ^]^ Ulcerative colitis (UC), a chronic, relapsing illness, which starts in the rectum and generally extends proximally in a continuous manner through part of, or the entire, colon, affecting millions of patients worldwide, is demonstrated to be strongly associated with microbial dysbiosis.^[^ ^8^, ^9^, ^10^, ^11^ ^]^ For example, *Faecalibacterium prausnitzii*, a kind of butyrate‐producing bacteria, is associated with the differentiation and expansion of Tregs as well as the growth of epithelial cells, was decreased in UC.^[^ ^12^, ^13^ ^]^ While the increase of sulfate‐reducing bacteria in UC would result in the production of hydrogen‐sulfate, consequently damaging the intestinal barrier and activating mucosal inflammation.^[^ ^14^ ^]^ *Bacteroides fragilis* can alleviate inflammation by promoting the development of regulatory T cells in UC.^[^ ^15^ ^]^ In addition, the activation of the farnesoid‐activated X receptor (FXR, a bile acid sensor) showed protection in UC by repression of nuclear factor‐κB (NF‐*κ*B) signaling. However, the bile salt hydrolase produced by intestinal bacteria is vital for the activation of FXR and dysbiosis could have a direct effect on FXR signaling.^[^ ^16^, ^17^, ^18^ ^]^ Probiotics have been proposed to modulate the composition of the gut microbiota and explored as promising strategies for UC treatment.^[^ ^19^, ^20^, ^21^, ^22^ ^]^ However, far from the ideal, probiotics are hard to efficiently exert their beneficial effects in disease prevention and treatment because they would suffer serious damage from gastric acid, digestive enzymes, or bile salts in the stomach and intestines, leading to massive death by oral medication.^[^ ^23^, ^24^, ^25^, ^26^ ^]^ In addition, the rapid gastrointestinal (GI) transit time contributes to the inability of probiotics to retain and colonize the gut.^[^ ^27^, ^28^ ^]^ To surmount these challenges, several methods such as capsules, tablets, and pellets have been developed. While these approaches have been successful in preventing chemical degradation by acid and enzymes, the short intestinal retention time has still not been tackled effectively.^[^ ^29^, ^30^ ^]^ Therefore, enhancing the survival of probiotic bacteria in extreme conditions while strengthening the proliferation and colonization is crucial for ensuring their optimal function as health‐promoting agents.

Surface decoration of bacteria is a simple yet efficient strategy, which not only gives bacteria the additional ability to resist environmental threats but also endows them with exogenous characteristics that are neither inherent nor naturally achievable in a programmed fashion.^[^ ^30^, ^31^, ^32^, ^33^ ^]^ Materials such as chitosan and alginate,^[^ ^28^ ^]^ mesoporous silica and carbon quantum dots,^[^ ^34^ ^]^ liposomes,^[^ ^27^ ^]^ silk fibroin,^[^ ^24^ ^]^ and tannic acid^[^ ^23^ ^]^ have been exploited to decorate the probiotics for protection from physiological GI condition and improving the therapeutic efficacy. However, the inhibition of intestinal inflammation during the probiotics delivery has not received much attention. The GI of UC is typically characterized by an imbalance of inflammatory homeostasis, which increases the local presence of reactive oxygen species (ROS), in turn, decreases the viability of probiotics and significantly affects colonization.^[^ ^23^, ^35^, ^36^ ^]^ Therefore, probiotics are often unable to retain and colonize the gut in pathological conditions like UC.^[^ ^23^, ^37^, ^38^ ^]^ The mucus layers in the small intestine, cecum, and colon are a crucial part of the epithelial defenses and are habited by intestinal microbes. However, the mucus layer in UC is characterized by destruction and further results in a vicious circle of microbial dysbiosis and colonic inflammation.^[^ ^39^ ^]^ Some studies have demonstrated that inflammation suppression helps to repair the mucus barrier and thus improves the structure of gut microbiota and enhances the retention of probiotics, which gives a welcome fillip to the UC treatment.^[^ ^40^, ^41^ ^]^ In addition, clinical data indicate that gut microbiota management in combination with anti‐inflammation administration is capable of improving the successful UC treatment.^[^ ^42^ ^]^ For clinical UC therapy, 5‐aminosalicylic acid (5‐ASA) is the predominant active moiety through local anti‐inflammation at the colonic lesion. However, 5‐ASA is rapidly absorbed in the upper GI tract by oral medication.^[^ ^43^, ^44^ ^]^ To further improve 5‐ASA release profiles in the colon, balsalazide (Bal), a 5‐ASA prodrug, which releases the active 5‐ASA only into the colon by colonic bacterial azo‐reductases with minimal systemic absorption and well toleration,^[^ ^45^, ^46^, ^47^ ^]^ is widely used to orchestrating the pathological niche for enhancing UC treatment.

**Scheme 1:**
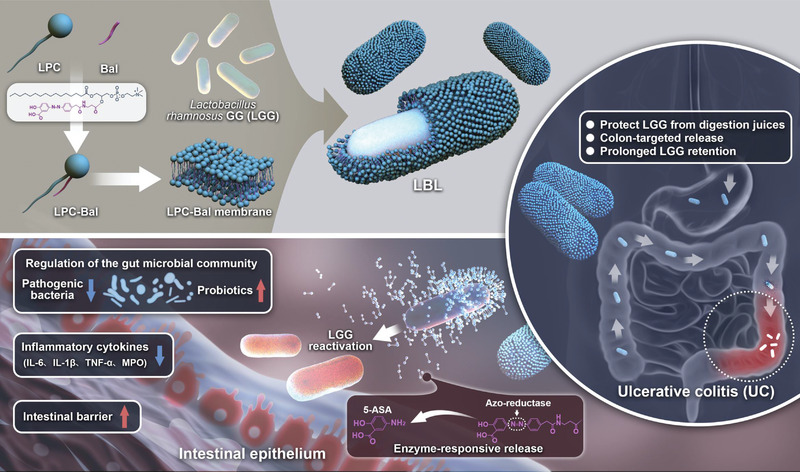
Schematic illustration of the LBL preparation process and the mechanism of LBL treatment of ulcerative colitis (UC).

Herein, we developed a simple and efficient method to construct the oral bioengineered probiotics, which can resist digestion in the whole GI, regulate the pathological environments, modulate the microbial composition, and consequently improve UC profiles (**Scheme** **1**). Especially, the coating composed of lipidic prodrug (LPC‐Bal) endows lactobacillus rhamnosus GG (LGG) with the capacity to resist digestion juices from the stomach and intestine to maintain its biological activity. After entering the colon, the prodrug release 5‐ASA under azo‐reductase to suppress the colonic inflammation and improve the pathological environment, thus, providing a favorable microenvironment for LGG colonization. Meanwhile, the LGG is able to efficiently colonize the intestine and subsequently modulate the gut microbiota for synergistically improving UC lesions.

## 2 Results and Discussion

**Figure 1:**
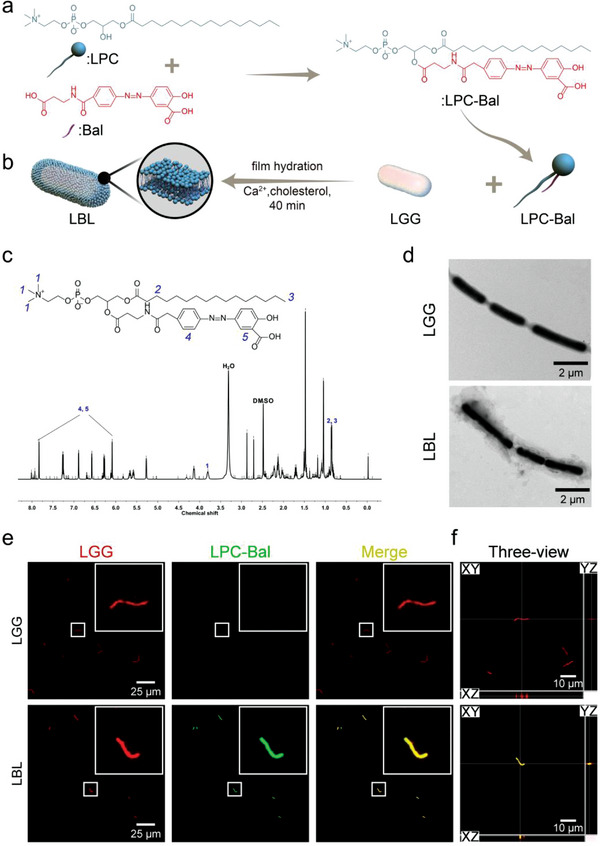
Fabrication and characterization of LBL. a) Schematic illustration of synthesis and the structure of LPC‐Bal. b) The fabrication process of LBL. c) 600 MHz ^1^H nuclear magnetic resonance spectroscopy (^1^H NMR) of LPC‐Bal. d) Representative TEM images and e) CLSM images of the native and decorated LGG. The coating and LGG were stained with FITC‐DSPE‐mPEG_2000_ and Cy5‐NHS, respectively. f) The *x–z* and *y–z* views of the fluorescence images processed by Imaris, demonstrating the colocalization of LGG and the coating.

Bal is conjugated to 1‐palmitoyl‐sn‐glycero‐3‐phosphocholine (LPC‐Bal) (**Figure** **1**a) and modified onto the surface of LGG via the interfacial supra‐molecular self‐assembly (Figure 1b) to construct an “Engineered probiotics” (LBL). LGG has proven to be able to enhance intestinal functional maturation and improve the composition of gut microbiota, thereby protecting against UC.^[^ ^48^, ^49^ ^]^ With the help of this approach, LBL is expected to retain the bioactivity after oral administration and specifically release drugs at colon lesions to improve pathological conditions for promoting colonization. To determine the successful synthesis of LPC‐Bal, ^1^H NMR (Figure 1c) and ^13^C NMR (Figure S1, Supporting Information) spectra were introduced to determine the characteristic peaks of LPC and Bal. The magnetic resonance peaks at the range of 6.00–8.00 ppm were ascribed to the hydrogen proton of the benzene ring (*4, 5*) for Bal. The magnetic resonance peak of —C*H*_3_ (*1*) was located at 3.79 ppm for LPC. Additionally, the magnetic resonance peaks were found at 0.89–1.00 ppm for —C*H*_2_— and —C*H*_3_ (*2, 3*) of LPC (Figure 1c). The magnetic resonance peaks at the range of 120.45–124.92 and 129.5–131.49 ppm were ascribed to the carbon nuclear of the benzene ring (*4, 5*) for Bal. The magnetic resonance peak of —*C*H_3_ (*1*) was located at 42.68–42.92 ppm for LPC. Additionally, the magnetic resonance peaks were found at 36.81 and 38.77 ppm for —*C*H_2_— and —*C*H_3_ (*2, 3*) of LPC (Figure S1, Supporting Information). The results of the typical magnetic resonance peaks demonstrated that LPC‐Bal was successfully synthesized. In addition, the molecular weight of Bal and LPC‐Bal were confirmed by mass spectrometry (MS). The MS revealed an exact molecular weight of 835.2619, which was in agreement with the theoretical molecular weight of LPC‐Bal (Figure S2, Supporting Information). The chemical binding or intermolecular bind interactions among the components was investigated by Fourier transform infrared microscopy (FT‐IR) (Figure S3, Supporting Information). In the spectrum of Bal, the peak of —OH or —NH— was found at 3061 cm^−1^, and the formation vibration peak of —NH— was observed at 1541 cm^−1^. Moreover, peaks of —CO—, —N=N—, C—H, and C=C appeared at 1697, 1450, 857, and 770 cm^−1^, respectively. The skeleton vibration peaks of the benzene ring were found at 1639 and 1636 cm^−1^. In the spectrum of LPC, the characteristic peak of —OH, C=O, P=O, and C—C appeared at 3143, 1735, 1175, and 718 cm^−1^, respectively. Meanwhile, the peaks of CH_3_—N^+^— and —CH_2_—N^+^— were found at 2849–2914 cm^−1^. After Bal was grafted on the LPC, the peaks of C=O, —OH were observed at 1725 and 3387 cm^−1^. Notability, the peaks of P=O, —NH— and —N=N— could be found at 1179, 1547, and 1450 cm^−1^, respectively. The peaks at 2849–2914 cm^−1^ were found for CH_3_—N^+^— and —CH_2_—N^+^—, respectively. The skeleton vibration peaks were found at 1639 and 1636 cm^−1^ for the benzene ring. Therefore, the above mentioned results confirmed that Bal was successfully grafted on the skeleton of LPC, which was consistent with the results of NMR analysis. The ultraviolet‐visible (UV–vis) spectra of LPC, Bal, and LPC‐Bal on Dimethyl sulfoxide (DMSO) (20 µg mL^−1^) were measured. LPC‐Bal exhibited characteristic peaks at ≈360 nm (Figure S4, Supporting Information).

Besides, the presence of the coating was confirmed by transmission electron microscopy (TEM). A well‐defined opaque shell boundary was observed on LBL, indicating that LGG was successfully wrapped with LPC‐Bal (Figure 1d). In addition, to further confirm the successful coating, LPC‐Bal and LGG were stained with fluorescein isothiocyanate 1,2‐distearoyl‐phosphatidylethanolamine‐methyl‐polyethyleneglycol conjugate‐2000 (FITC‐DSPE‐mPEG_2000_) and Cy5‐*N*‐hydroxysuccinimide (NHS), respectively. Under confocal laser scanning microscope (CLSM) imaging, the red fluorescent LGG and green fluorescent LPC‐Bal coating showed a high overlap (Figure 1e). Meanwhile, the orthogonal projection in *x–z* and *y–z* views processed by Imaris were shown to confirm the desirable colocalization of LGG and the coating (Figure 1f). Flow cytometric analysis further revealed a significant increase in the mean fluorescent intensity over the uncoated LGG (Figure S5, Supporting Information). Similarly, the particle size (Figure S6, Supporting Information) increased from 1836 ± 128 to 1919 ± 109 nm, and the zeta potential (Figure S7, Supporting Information) decreased from −12.9 ± 1.6 to −6.8 ± 0.3 mV after coating, further verifying the successful encapsulation. The loading efficiency of Bal on LBL was calculated according to the standard curve. From the results shown in Figure S8 in the Supporting Information, the loading efficiency was ≈3.76 mg of Bal per 1 × 10^9^ CFU LBL. The free Bal administration was the equal equivalent of the Bal content in LBL group.

**Figure 2:**
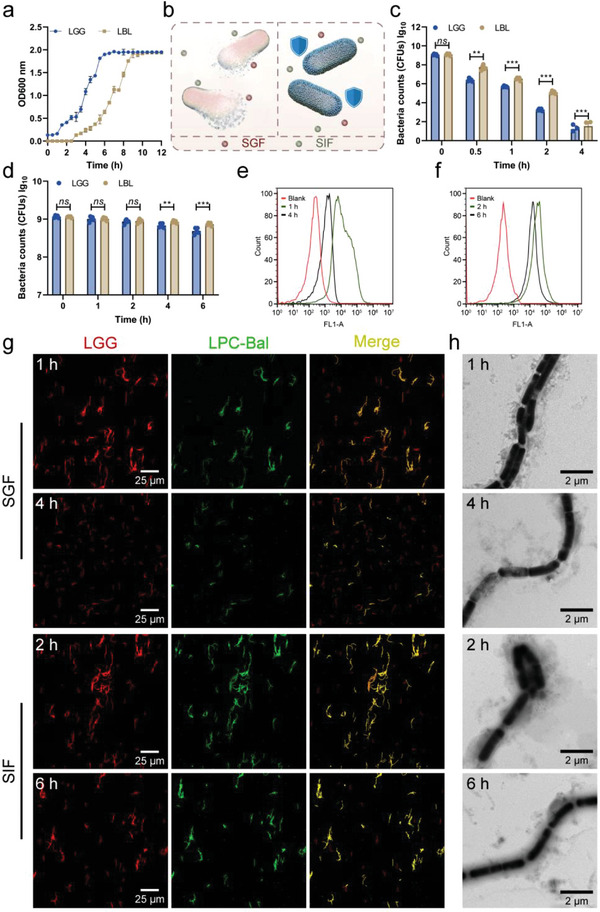
In vitro resistance of LBL to the simulated harsh environments. a) Growth curves of the native and decorated LGG in MRS culture medium at 37 °C and the OD600 was recorded every 30 min for 12 h by a microplate reader (*n* = 3). b) Schematic representation of the in vitro resistance of LBL against environmental assaults. Survival of the native LGG and LBL after incubation with c) the simulated gastric fluid (SGF) or d) the simulated intestinal fluid (SIF) at 37 °C. The number of the survived bacteria was quantified by spreading 100 µL of the serially diluted samples of each suspension onto MRS agar plates and incubated at 37 °C for 24 h (*n* = 5). Flow cytometric analysis of LBL after exposure to e) SGF or f) SIF. The coating was stained with FITC‐DSPE‐mPEG_2000_. g) CLSM images of LBL after incubation with SGF or SIF for the predetermined time interval. The coating and LGG were stained with FITC‐DSPE‐mPEG_2000_ and Cy5‐NHS, respectively. h) Representative TEM images of LBL after SGF or SIF treatment for predefined time points. Data were shown as mean ± SD. Unpaired *t* test (two‐tailed) was performed on (c) and (d), *ns* (no significance), *P* < 0.01 (**), or *P* < 0.001 (***).

The viability and growth of the LBL were monitored to validate the protective effects of the coating on LGG. It was evident from the results that the uncoated LGG proliferated rapidly, while LBL presented proliferation inhibition for ≈2 h (**Figure** **2**a). However, both the uncoated and coated LGG had similar growth rates and eventually reached similar quantities, demonstrating that the coating did not significantly affect the ultimate viability of LGG. As for the 2 h growth arrest, it should result from the coating shielding the exchange of matter and energy between LGG and the environment. Then, the protective effect against the simulated gastric fluid (SGF) or simulated intestinal fluid (SIF) was verified (Figure 2b). LGG and LBL with an equal amount were respectively incubated with SGF or SIF at 37 °C, and then, the colonies were counted at a predetermined time interval. As reflected by the results, the acidic conditions induced the massive bacteria death, and the complete bacteria death occurred on LGG within 4 h incubation. However, LBL exhibited a favorable resistance to gastric acid, which was attributed to the presence of the shell shield (Figure 2c). A similar trend was also observed in the incubation of SIF. The results demonstrated that LGG inherently possessed a certain degree of resistance ability against the intestinal digestive fluid as a significant difference was detected until 2 h, while the difference gradually increased with the extension of incubation time (Figure 2d). Following 6 h exposure to SIF, LBL resulted in the 1.6‐time more survival than that of the undecorated LGG. To further confirm that the enhanced resistance was attributed to the presence of the coating, flow cytometry was analyzed. The coating was stable as the fluorescent signals could be detected even incubated in SGF for up to 4 h, which exceeded the gastric retention time of mice (Figure 2e). A little change to a lower fluorescence intensity occurred from 2 to 6 h in SIF, reemphasizing the stability of the coating (Figure 2f). Furthermore, CLSM images showed that with the extension of incubation time, the coating had fallen off from the bacteria, but some of the bacteria were still colocated with the coating (Figure 2g). TEM images visually demonstrated the morphology of the LBL, while some bacteria appeared aberrant with the absence of the coating, and some bacteria was still been wrapped with the opaque shell even at 4 h in SGF (Figure 2h). Taken together, these results indicated that the coating was stable under gastric acid and intestinal digestive fluid conditions, demonstrating the feasible and efficient approach for probiotic protection.

**Figure 3:**
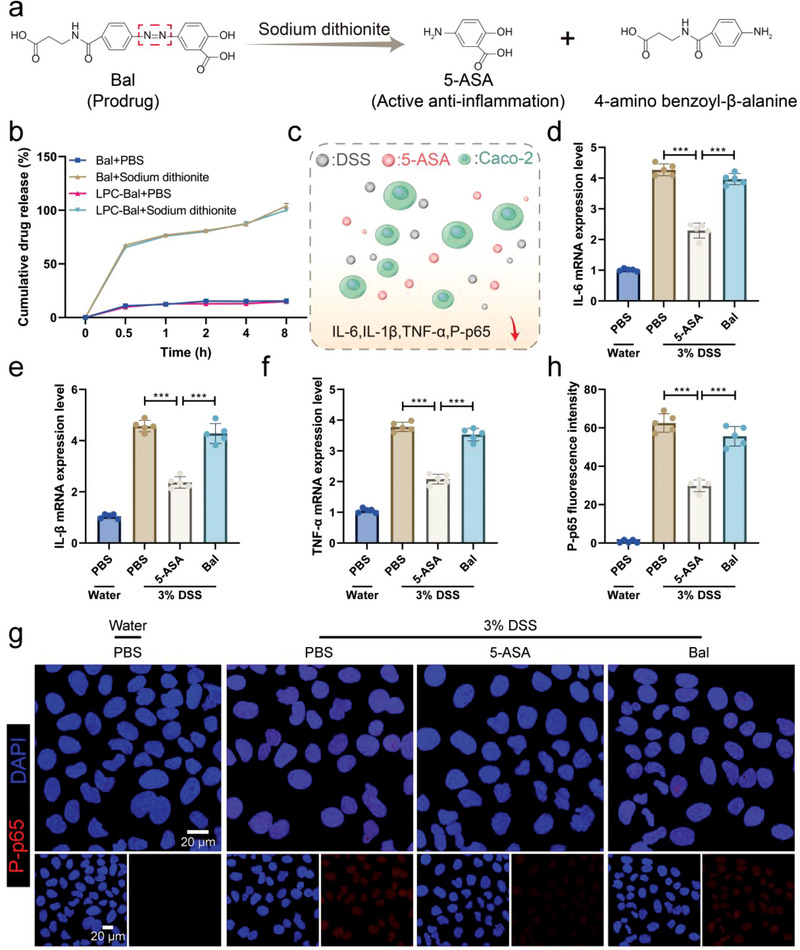
The anti‐inflammation potential of the coating. a) The release kinetics of balsalazide (Bal) to 5‐aminosalicylic acid (5‐ASA) under sodium dithionite. b) The cumulative release curve of Bal and LPC‐Bal with or without sodium dithionite (*n* = 9). c) Schematic diagram of inflammation inhibition effect of 5‐ASA in Caco‐2 cells induced by DSS. d–f) Total RNA was extracted from Caco‐2 cells. The mRNA expression levels of IL‐6, IL‐1*β*, and TNF‐*α* were determined by qPCR. g) Representative immunofluorescence staining and h) quantitative analysis for TLR4/NF‐kB signaling pathway‐related protein P‐p65 (*n* = 5). Data are shown as mean ± SD. Significances were determined by one‐way analysis of variance (ANOVA), followed by post hoc pairwise comparisons with the Tukey honest significant difference *P* < 0.001 (***).

Besides, the anti‐inflammatory potential of Bal, the main anti‐inflammatory activity cytokine of the coating, was further investigated. The release kinetics of Bal to 5‐ASA under sodium dithionite, a kind of reducing agent to mimic the azo‐reductase enzyme,^[^ ^50^, ^51^, ^52^ ^]^ was evaluated first (**Figure** **3**a). The cumulative release curve of Bal and LPC‐Bal showed that 5‐ASA was rapidly released within 0.5 h and completely within 8 h in the presence of sodium dithionite (2 mm). However, only 15% of the loaded drug was released in phosphate buffer (PBS) at the end of the experiment investigation (Figure 3b). In addition, the release kinetics of Bal and LPC‐Bal under SGF or SIF were also determined and no dramatic changes were observed (Figures S9 and S10, Supporting Information). For the cell compatibility study, the cytotoxicity of Bal and LPC‐Bal to Caco‐2 cells was investigated. The Caco‐2 cells were incubated with Bal or LPC‐Bal at the doses of 0.035, 0.07, 0.14, and 0.28 µmol mL^−1^ for 12 h. As shown in Figure S11 in the Supporting Information, favorable cell viability was detected even the drug concentration reached 0.28 µmol mL^−1^ and no significant differences were found between Bal and LPC‐Bal. Immediately afterward, the in vitro anti‐inflammatory potential of Bal in terms of cell cytotoxicity of Caco‐2 cells induced by dextran sulfate sodium (DSS) was performed (Figure 3c). Caco‐2 cells are the human colon epithelial cancer cell line, which can express surface markers and tight junctions characteristic of the intestinal villus, and have been widely used as a model in mechanistic studies. The relative mRNA expressions of the inflammatory cytokines interleukin‐6 (IL‐6), interleukin‐1β (IL‐1*β*), and tumor necrosis factor‐α (TNF‐*α*) in Caco‐2 cells were detected with quantitative real‐time polymerase chain reaction (qPCR). The expression levels of these indices significantly increased after incubation with DSS, while 5‐ASA exhibited the significant inflammation inhibitory effects (Figure 3d–f). In addition, the expression level of P‐p65, displayed by immunofluorescent staining, was also decreased after treated with 5‐ASA (Figure 3g,h). Similarly, the contents of IL‐6, IL‐1*β*, and TNF‐*α* in DSS‐incubated macrophage model cells (RAW 264.7), analyzed using enzyme‐linked immunosorbent assay (ELISA), were also decreased with 5‐ASA treatment (Figure S12, Supporting Information). Together, Bal could timely release 5‐ASA in response to azo‐reductase to regulate inflammation at the cellular level, indicating the anti‐inflammation potential of the functional coating.

**Figure 4:**
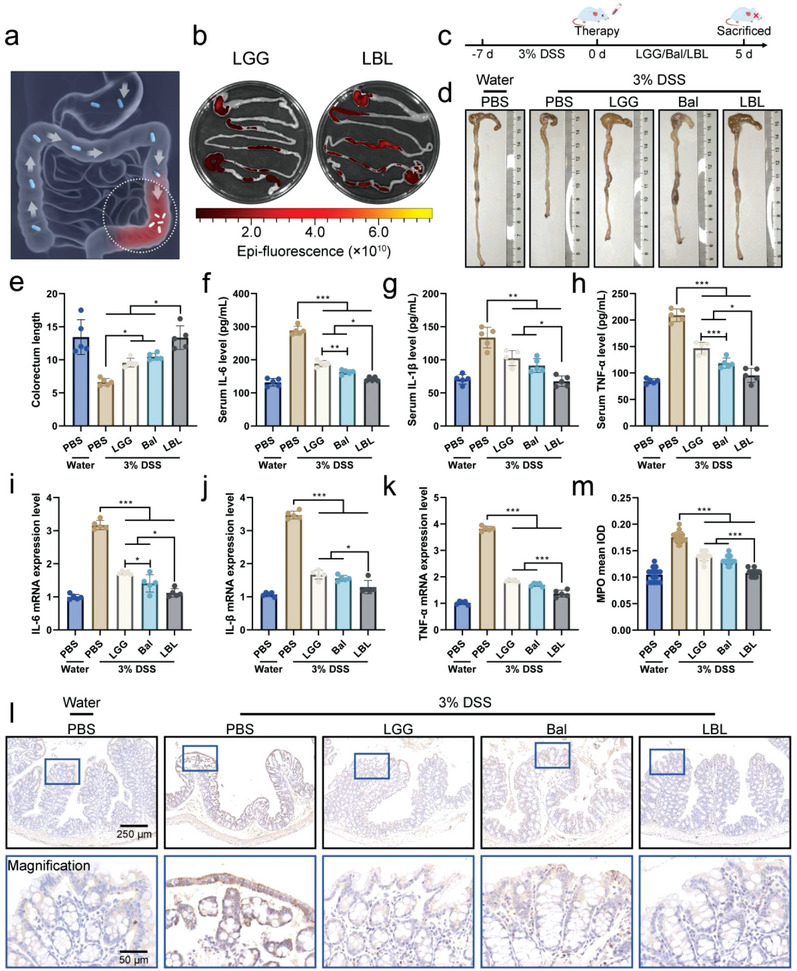
In vivo treatment of LBL against ulcerative colitis (UC). a) Schematic diagram of the treatment of LBL on UC. b) Representative IVIS images of the GI tract 4 h postadministration of the native and decorated LGG. c) Illustration of the experimental protocol. Mice were given drinking water containing 3% DSS for 7 d to induce colitis followed by administration with LBL for 5 d. d) Representative images of the harvested intestinal tracts. e) Length of the colorectum (*n* = 5). f–h) Serum levels of IL‐6, IL‐1*β*, and TNF‐*α* (*n* = 5). i–k) Total RNA was extracted from the colon (*n* = 5). The mRNA expression levels of IL‐6, IL‐1*β*, and TNF‐*α* were determined by qPCR. l) Representative images of myeloperoxidase (MPO) immunohistochemistry of the colon. m) The mean integrated optical density (IOD) of MPO, quantified by Image J (*n* = 15). Data are shown as mean ± SD. Significances were determined by one‐way ANOVA, followed by post hoc pairwise comparisons with the Tukey honest significant difference. *P* < 0.05 (*), *P* < 0.01 (**), or *P* < 0.001 (***).

To assess the potential functions of the coating to increase the resistance of GI environment and enhance the colonization of LGG in vivo, the reservation of the decorated and undecorated LGG stained by Cy5‐NHS was detected by In Vivo Imaging System (IVIS) Spectrum at the predetermined time interval. LGG and LBL with equal amounts (1 × 10^9^ CFU) were administrated orally. LBL showed a higher fluorescence intensity than that of LGG at 4 h (**Figure** **4**a,b). The fluorescence intensity in the stomach, intestine, cecum, and colorectum was quantified (Figure S13, Supporting Information). The varying results may be ascribed to the differences in the integrity of the bacteria as the coating that could protect the bacteria from the destruction of digestive juice. The above results suggested that the coating could greatly improve the bacterial bioactivity and colonization rate. After determining the protective effect and enhanced colonization ability of the coating, the treatment effect of LBL on UC induced by DSS was assessed. Mice were treated with 3% DSS in the drinking water for 7 d to induce colitis followed by oral administration treatment for 5 d (Figure 4c). Survival was tracked during the experimental period, death occurred from the second day of the DSS‐induction phase, and with the intervention of various formulations, the death rate was contained (Figure S14, Supporting Information). The final body weight was recorded, and a sharp loss was noted in the control group with the DSS treated mice, while LBL treated group displayed a significantly improved recovered performance (Figure S15, Supporting Information). The length of the colorectum after 5 d post‐treatment was measured, and LBL treat group showed significant protection against the reduction of the length induced by the DSS treated group (Figure 4d,e). Moreover, the therapeutic effect of LBL in UC was assessed from the aspects of inflammation and intestinal barrier integrity. The levels of pro‐inflammatory cytokines including IL‐6, IL‐1*β*, and TNF‐*α* in the serum were measured with ELISA. LBL could significantly reduce the levels of these inflammatory factors to the comparable levels of healthy control mice, and Bal exerted a better inflammation inhibitory effect than LGG (Figure 4f–h). The inflammation in the colon reflected by expression levels of inflammation‐related factors detected by qPCR and immunohistochemistry was attenuated after treatment of LBL. As shown in Figure 4i–k, compared with the DSS treated group, LBL treated group apparently reduced the expression of IL‐6, IL‐1*β*, and TNF‐*α* by ≈60%, respectively. Specifically, the IL‐6 mRNA expression in Bal treated group was lower than that of the LGG treated group, indicating the efficient inflammation‐inhibition effect of Bal. Furthermore, the mean integrated optical density (IOD) of myeloperoxidase (MPO), a marker of oxidative stress and inflammation,^[^ ^53^ ^]^ was significantly decreased in DSS‐induced mice after being administered with LBL (Figure 4l,m). Superoxide dismutase (SOD) has been reported to effectively clear reactive oxygen species and relieve inflammation by catalyzing the breakdown of the superoxide anion free radicals into hydrogen peroxide and molecular oxygen.^[^ ^54^, ^55^ ^]^ Malondialdehyde (MDA) is the marker of lipid peroxidation, the increase of superoxide anion free radicals can lead to the formation of MDA, which induces cell death and further results in tissue injury and inflammation.^[^ ^56^ ^]^ Therefore, the levels of SOD and MDA were analyzed with ELISA as oxidative stress indicators. LBL exhibited favorable antioxidant effects (Figure S16, Supporting Information).

**Figure 5:**
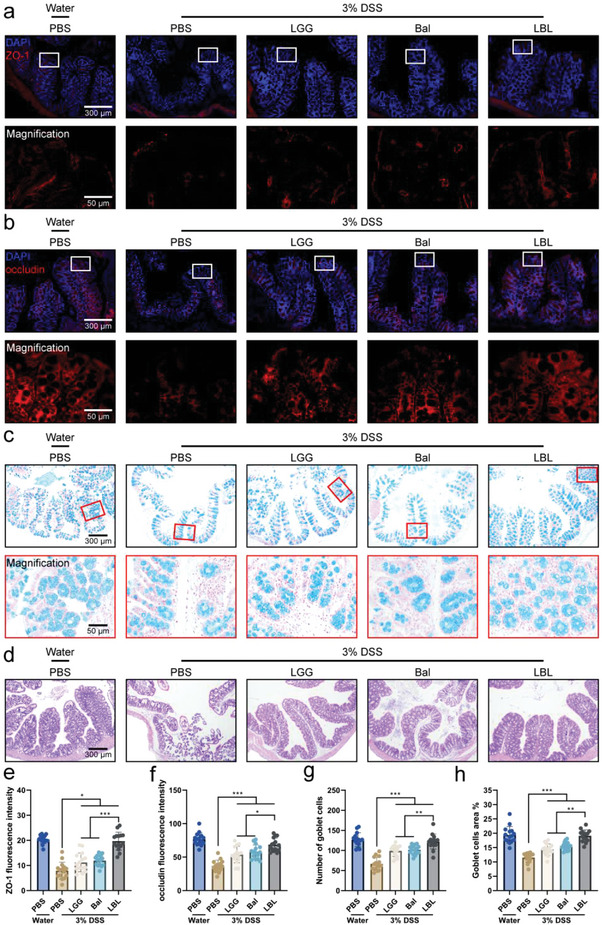
Improvement of the gut barrier after administration of LBL. a) Representative immunofluorescence staining for zona occludens protein‐1 (ZO‐1) of colonic tissue. b) Representative immunofluorescence staining for occludin of colonic tissue. c) Representative photoimages of alcian blue staining of colonic tissue. d) Representative images of H&E in mouse colon tissue. e,f) The fluorescence intensities of ZO‐1 and occludin were measured by Image J (*n* = 15). g,h) The number and the area of goblet cells were calculated with Image J (*n* = 15). Data are shown as mean ± SD. Significances were determined by one‐way ANOVA, followed by post hoc pairwise comparisons with the Tukey honest significant difference. *P* < 0.05 (*), *P* < 0.01 (**), or *P* < 0.001 (***).

Furthermore, intestinal permeability was a functional feature of the intestinal barrier, and dysfunction of the intestinal barrier could lead to increased intestinal permeability and intestinal inflammatory disease.^[^ ^57^ ^]^ Therefore, the expression levels of antizona occludens protein‐1 (ZO‐1) and occludin, as the integrity indicator of the tight junctions, were detected. A significant decrease occurred in the DSS treated group, however, LBL could reverse this trend (**Figure** **5**a,b,e,f). Mucus produced by goblet cells in the epithelium was a critical barrier in the gut, while, UC was characterized by goblet cell depletion.^[^ ^58^ ^]^ Consequently, the goblet cell density was assessed through alcian blue staining. Consistent with the results of ZO‐1 and occludin, DSS treated group considerably decreased the number of goblet cells, but the damaging effect was significantly attenuated after LBL administration (Figure 5c,g,h). Histological sections of the colon stained with hematoxylin and eosin disclosed the integrated and well‐preserved histoarchitecture in healthy control mice. Although DSS would lead to intestinal villi disruption, visible damage was not observed in LBL treated mice, conversely, protection against alteration of colonic epithelial integrity (Figure 5d).

**Figure 6:**
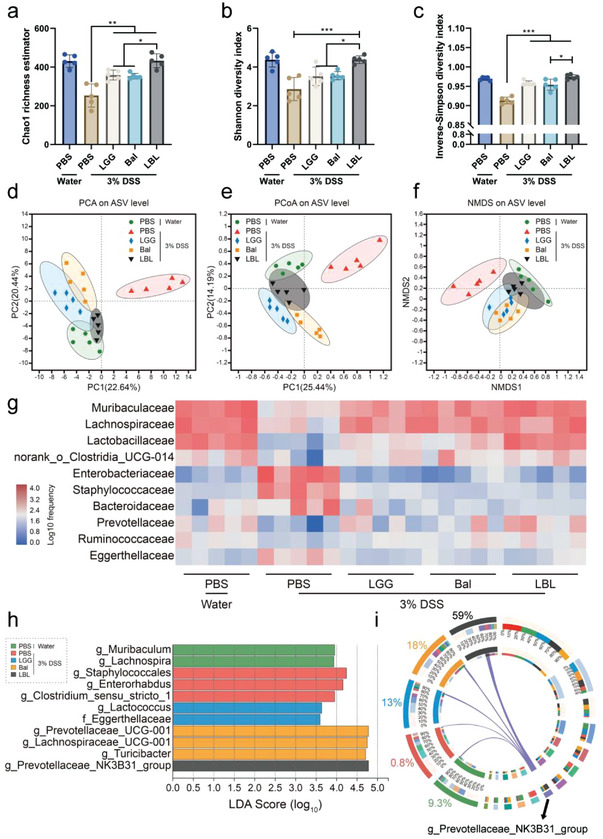
Regulation of LBL on the gut microbial community. The V3‐V4 region of the bacterial 16S rRNA was sequenced in fecal samples and comparisons were carried out using alpha and beta diversity. a) Chao1, b) Shannon, c) Inverse‐Simpson, d) principal components analysis (PCA), e) principal coordinate analysis (PCoA), and f) nonmetric multidimensional scaling (NMDS) indices of ASV level. g) Heatmap of the ten most abundant taxa at the family level. h) LEfSe histograms. Bacterial taxa that met the criterion of an LDA score >3.5 were considered biomarker taxa. i) Circos diagram to reflect the distribution proportion of the dominant specie in different groups. Data are shown as mean ± SD (*n* = 5). Significances were determined by one‐way ANOVA, followed by post hoc pairwise comparisons with the Tukey honest significant difference. *P* < 0.05 (*), *P* < 0.01 (**), or *P* < 0.001 (***).

Gut microbiota imbalance has been suggested to play a vital role in UC. Therefore, the impact of LBL on the community structure of the gut microbiota was analyzed. Amplicon sequence variants (ASV) were analyzed using Qiime2 to calculate multiple diversity metrics. Alpha‐diversity indices, including the Chao 1, Shannon, and inverse Simpson indices were significantly increased in LBL administration mice, suggesting that LBL could positively regulate the gut microbiota structure (**Figure** **6**a–c). Beta‐diversity was evaluated using principal components analysis (PCA), principal coordinate analysis (PCoA), and nonmetric multidimensional scaling (NMDS) to explore and visualize similarities or dissimilarities of the gut microbiota. A clear separation was revealed between the PBS + 3% DSS treated group and other treated groups indicating that the gut microbiota composition of PBS + 3% DSS was significantly changed under the influence of DSS. However, the ellipses of normal control and bacterial/drug intervention groups overlapped and no individuals fell outside the ellipses, demonstrating that the microbial community structures of these four groups were partially similar. Moreover, LBL and normal control were spatially close to each other, suggesting that LBL could positively regulate the gut microbiota structure and be much more effective than the single strategy ones (Figure 6d–f). The detailed changes in the gut microbes were analyzed and visualized using the heatmap. The mean relative abundance of the ten most abundant taxa at the family level were presented (Figure 6g), and the corresponding statistical analysis were performed (Figure S17, Supporting Information). Results showed that LBL treatment significantly increased the abundance of *Muribaculaceae*, *Lachnospiraceae*, *Lactobacillaceae*, *norank_o_Clostridia_UCG‐014*, *Prevotellaceae*, and *Ruminococcaceae*, and simultaneously reduced the abundance of *Enterobacteriaceae*, *Staphylococcaceae*, and *Eggerthellaceae*. Previous studies showed that *Lachnospiraceae* and *Ruminococcaceae* were negatively associated with DSS‐induced UC.^[^ ^59^, ^60^ ^]^ Additionally, it has been reported that *Lachnospiraceae* and *Ruminococcaceae* could impact their hosts by producing short‐chain fatty acids, converting primary to secondary bile acids, and facilitating colonization resistance against intestinal pathogens.^[^ ^10^, ^61^ ^]^ Lots of *Lactobacillus* belonging to *Lactobacillaceae* were established to play important roles in the treatment of UC.^[^ ^20^, ^62^, ^63^, ^64^ ^]^ *Prevotellaceae* and *norank_o_Clostridia_UCG‐014* were considered to be beneficial for UC and negatively correlated with inflammation.^[^ ^65^, ^66^, ^67^, ^68^ ^]^ In combination, these results further confirmed that LBL could positively modulate the gut microbial community structure. The linear discriminant analysis (LDA) effect size (LEfSe) analysis with an LDA score 3.5 was then performed to identify significant biomarkers at the genus level (Figure 6h). The normal control group and bacteria/drug intervention groups were mainly characterized by beneficial bacteria. The *g_Prevotellaceae_NK3B31_group* was a kind of butyric acid‐producing bacteria and had been considered to be beneficial bacteria.^[^ ^69^, ^70^ ^]^ The proportion of *g_Prevotellaceae_NK3B31_group* in each group was presented with a Circos figure and LBL had the highest proportion (59%) (Figure 6i). Together, these results highlighted the fact that LBL could remarkably restore the imbalanced gut microbiota in UC by increasing the beneficial bacteria and reducing pathogenic bacteria.

## 3 Conclusion

In summary, a bioengineered probiotic decorated with a multifunctional coating was constructed and proved to be able to improve the UC in mice induced with DSS. It has been demonstrated in vitro that the coating could maintain bacterial biological activity by protecting LGG from the attack of SGF and SIF. In addition, the coating possessed an excellent anti‐inflammatory capacity proved in Caco‐2 cells. In vivo results further demonstrated that compared with the native LGG, LBL exhibited enhanced intestinal colonization. Moreover, the levels of systemic and colonic tissue inflammation were significantly decreased, and the damaged intestinal barrier function and dysregulated gut microbiota were improved after being administered with LBL. These results demonstrated that LBL with the synergistically combined benefits of probiotics and drugs could be a promising candidate for feasible and efficient UC management.

## 4 Experimental Section

### Materials and Strains

*Lactobacillus rhamnosus* ATCC53103 (LGG) was purchased from Guangdong Microbial Culture Collection Center (Guangdong, China, CHN) and grown in an de Man, Rogosa, Sharpe (MRS) medium at 37 °C. Caco‐2 cells, human intestinal epithelial cells, were purchased from the American Type Culture Collection (ATCC) and cultured in Dulbecco's modified Eagle's medium. LPC, Bal, FITC, and Cy5 were purchased from Macklin (Shanghai, CHN). SGF containing pepsin (3.2 g) and NaCl (2.0 g) in deionized water (1000 mL) was adjusted to pH 2.0 with HCl and then filtered by a 0.22 µm membrane. SIF containing KH_2_PO_4_ (6.8 g), bile salts (3 g), and trypsin (10 g) was adjusted to pH 6.8 with NaOH and then filtered by a 0.22 µm membrane.

### Acidification of Balsalazide Disodium

Balsalazide disodium (100 mg) was dissolved in ultrapure water (1 mL), followed by the dropwise addition of HCl solution (10 mL, pH 1.0), and Bal will be precipitated from the solution. The sample was then centrifuged and washed with HCl (pH 1.0) twice. Purified Bal was obtained by lyophilization.

### Preparation and Characterization of LPC‐Bal

Bal, 4‐dimethylamino‐pyridine, and NHS were dissolved in *N*,*N*‐dimethylformamide (DMF) at a 1: 1.2: 1.2 molar ratio and reacted for 4 h at 40 °C with stirring. Then, LPC with an equal molar mass of Bal has added subsequently for the next 8 h. Afterward, the resultant solution was dialyzed with ultrapure water using a dialysis bag with a cutoff of 500 Da for 8 h, and water was exchanged at 2 h internals. Purified LPC‐Bal was obtained by lyophilization. LPC‐Bal was dissolved in DMSO‐d6, and then 400 µL solution was transferred into NMR tubes. ^1^H NMR (10 mg) and ^13^C NMR (50 mg) measurements were made on Bruker AVANCE II 600 spectrometer at 600 MHz and Bruker 700 M spectrometer at 700 MHz respectively. MS (10 mg) was performed in negative electrospray ionization mode on Waters G2‐XS QTOf mass spectrometer. FT‐IR spectra were recorded on Nicolet iS50 FT‐IR with the KBr pellet technique (2 mg of sample in 200 mg of KBr). The UV–vis absorption spectra were recorded with a 300–800 nm scan (DU730, Beckman Coulter).

### Preparation of FITC‐DSPE‐mPEG_2000_ and Cy5‐NHS

FITC and DSPE‐mPEG_2000_ were dissolved in DMF and stirred overnight at a 1.2: 1 molar ratio at room temperature. The solution was purified by dialysis bag with a 2000 Da cutoff in ultrapure water for 8 h protected from light. FITC‐DSPE‐mPEG_2000_ was obtained via lyophilization. The FITC‐labeled coating on LGG was fabricated via co‐assembly of FITC‐DSPE‐mPEG_2000_ and LPC‐Bal.

Cy5, 1‐ethyl‐3‐(3‐dimethylaminopropyl) carbodiimide hydrochloride, and NHS were dissolved in ultrapure water at a 1: 1.2: 1.2 molar ratio and reacted for 8 h at 40 °C with stirring. The obtained solution was stored at −20 °C until further use.

### Preparation of LBL

LBL was prepared according to the previous study with minor modifications.^[^ ^27^ ^]^ Briefly, 1 × 10^9^ CFU of LGG was resuspended in ice‐cold (1 mL) calcium chloride solution (12.5 mm). LPC‐Bal (16.7 mg) and cholesterol (2.26 mg) were dissolved in chloroform (1 mL) and then evaporated by rotary evaporation at 37 °C to create a dry film. The obtained film was hydrated in bacterial suspension (1 mL) and vortexed for 40 min and then centrifuged at 3000 rpm to remove supernatant and resuspended in PBS (200 µL).

To calculate the drug loading of Bal in LBL, the LBL powder was dissolved in DMSO and the absorbance of gradient dilutions at 360 nm was measured using an ultraviolet spectrophotometer. The drug loading could be calculated according to the pre‐established standard curve of Bal.

The coating was stained by co‐assembly of LPC and FITC‐DSPE‐mPEG_2000_ (10% molar ratio to LPC).^[^ ^27^ ^]^ 1 × 10^9^ CFU of LGG in PBS (200 µL) was mixed with Cy5‐NHS (0.02 mg).

### Characterization of LBL

The morphology of LBL was visualized using TEM (ThermoFisher Scientific,Netherlands). LBL was resuspended into the water and the solution (10 µL) was dripped on the surface of a formvar/carbon‐coated copper grid (200 mesh) and dried at room temperature. The average size, size distribution, and zeta potential of LBL were evaluated using dynamic light scattering (Malvern Zetasizeer nano ZS, Great Britain). Colocalization analysis of LPC‐Bal and LGG was performed by CLSM (Leica, Germany).

### Growth Curves of LBL

The native and decorated LGG were prepared as described in the section of preparation of LBL. The native LGG and LBL were diluted with ice‐cold calcium chloride solution to an OD600 of 0.1, then the suspensions (10 µL) were added to the 96‐well plates with fresh MRS medium (200 µL) and incubated at 37 °C with slow shaking. OD600 measurements were taken every following 30 min for 12 h by a microplate reader (BioTek, USA).

### Cell Viability and Cytokine Assay

Caco‐2 cell viability was determined by cell counting kit‐8 (CCK‐8) assay (Beyotime, CHN). Caco‐2 cells were seeded at 96‐well plates in Dulbecco's modified Eagle's medium with GlutaMax and high glucose supplemented with 10% heat‐inactivated fetal bovine serum (Gibco, CHN) penicillin (100 U mL^−1^; Gibico, CHN), streptomycin (100 µg mL^−1^; Gibico, CHN), and 1 mm sodium pyruvate (Gibco, CHN) at 37 °C and 5% CO_2_ to form a confluent, quiescent monolayer. After incubation with different concentrations of Bal or LPC‐Bal for 12 h, 20 µL of CCK‐8 solution was added to each well, and the plate was incubated for an additional 2 h, followed by the measurement at 450 nm with a microplate reader.

For the anti‐inflammation experiment, the samples were processed as follows: a) Caco‐2 cells cocultured with PBS (100 µL mL^−1^); b) Caco‐2 cells cocultured with 3% DSS (100 µL mL^−1^). c) Caco‐2 cells cocultured with 3% DSS (100 µL mL^−1^) and 5‐ASA (0.65 µmol mL^−1^). d) Caco‐2 cells cocultured with 3% DSS (100 µL mL^−1^) and Bal (0.65 µmol mL^−1^). The expression levels of IL‐6, IL‐1*β*, and TNF‐*α* in the Caco‐2 cells for individual groups were assayed by ELISA according to standard procedures (R&D, USA).

### Total RNA Isolation and Quantitative Real‐Time PCR

Total RNA of Caco‐2 cells and colon was extracted with RNAiso reagent (Cat #: 9109, Takara‐Bio, CHN). Complementary DNA was synthesized using a complementary DNA synthesis kit (Cat #: RR047A, Takara‐Bio). qPCR was performed on the BioRad CFX Connect apparatus with a TB Green‐based real‐time PCR master mix (Cat #: RR820A, Takara‐Bio). The primers for each specific gene are listed in Table S1 in the Supporting Information.

### Resistance Assay In Vitro

A hundred microliters with an equal amount of native or decorated LGG were coincubated with SGF (900 µL) or SIF (900 µL) at 37 °C with gently shaking. 100 µL of each sample was taken, washed with fresh MRS medium, and spread on MRS agar plates at predetermined time points. The number of bacterial colonies was counted after 24 h incubation at 37 °C.

### Establishment of Colitis Models

Seven‐week‐old male C57BL/6 mice were obtained from GemPharmatech Co. Ltd and housed in a specific pathogen‐free animal facility. All of the animal care and experimental protocols were carried out with the approval of the Institutional Animal Care and Use Committee (IACUC) of Chongqing University (IACUC Issue No.: CQU‐IACUC‐RE‐202109–002). The mice were acclimated for one week before the experiments. Eight‐week‐old mice were fed with a normal chow diet ad libitum under a strict 12 h light/12 h dark cycle throughout the experimental session. The mice were randomly divided into six groups: a) mice in the control group were treated with sterile drinking water for 7 d and gavage with PBS for the additional 5 d; b) mice in the control group were treated with 3% DSS in the sterile drinking water for 7 d and gavage with PBS for the additional 5 d; c) mice were treated with 3% DSS in the sterile drinking water for 7 d and gavage with Bal (3.76 mg) for the additional 5 d; d) mice were treated with 3% DSS in the sterile drinking water for 7 d and gavage with LGG (1 × 10^9^ CFU) for the additional 5 d; e) mice were treated with 3% DSS in the sterile drinking water for 7 d and gavage with LBL (1 × 10^9^ CFU) for the additional 5 d.

### In Vivo Distribution of LBL

Eight‐week‐old male mice were overnight fasted and then gavaged with 1 × 10^9^ CFU of stained LBL. The mice were sacrificed at 4 h postadministered, and the GI tracts including stomach, intestine, cecum, and colon were imaged with IVIS Spectrum and the fluorescent signals of each part were analyzed by Living Image 4.5.5 (PerkinElmer, USA).

### Histopathology Analysis

Colon tissues were harvested and colon length was measured. All of the tissue samples were fixed in 3% paraformaldehyde and embedded in paraffin, followed by sectioning into 4 µm slices. Hematoxylin and eosin (H&E) staining was performed to evaluate the pathological progress. Alcian blue staining was used to detect mucus accumulation and identify goblet cells in the colon. For immunofluorescent staining, the section of the colon was treated with ZO‐1 or antioccludin at 4 °C, then rinsed and incubated with FITC‐conjugated secondary antibodies and counterstained with 4',6‐diamidino‐2‐phenylindole. For immunohistochemistry, the colonic tissue sections were incubated with an anti‐MPO antibody and then incubated with a horseradish peroxidase‐conjugated secondary antibody.

### Gut Microbiota Sequencing

Cecal contents were collected and the V3‐V4 (338F‐806R) region of the 16S rRNA gene from the fecal matter was sequenced using an Illumina Miseq platform (Majorbio, CHN) according to the manufacturer's protocol. The principal coordinate analysis (PCoA) and the NMDS of the microbiota were performed based on the Bray‐Curtis similarities.

### Statistical Analysis

All experiments were randomized and blinded. Statistical analyses were performed using Prism (Graphpad v9) and data were presented as mean ± standard deviation(SD). Statistical significance was determined at *P* < 0.05 through a one‐way analysis of variance with Tukey's/Dunn's multiple comparison test.

## Conflict of Interest

The authors declare no conflict of interest.

## Supporting information

Supporting Information

## Data Availability

The data that support the findings of this study are available from the corresponding author upon reasonable request.
