# Supplementary material for: Prodrug Integrated Envelope on Probiotics to Enhance Target Therapy for Ulcerative Colitis
Source: Adv Sci (Weinh). 2022 Dec 11;10(4):2205422. doi: 10.1002/advs.202205422 (PMC9896077; doi:10.1002/advs.202205422)
Supplement: Supplementary file 1 — Supporting Information [file ADVS-10-2205422-s001.pdf]

ADVANCED SCIENCE
Open Access

Supporting Information

for *Adv. Sci.*, DOI 10.1002/advs.202205422

Prodrug Integrated Envelope on Probiotics to Enhance Target Therapy for Ulcerative Colitis

Kun Zhang, Li Zhu, Yuan Zhong, Lixin Xu, Chunhui Lang, Jian Chen, Fei Yan, Jiawei Li, Juhui Qiu, Yidan Chen, Da Sun, Guixue Wang, Kai Qu, Xian Qin* and Wei Wu**

Supporting Information

Prodrug integrated envelope on probiotics to enhance target therapy for ulcerative colitis

Kun Zhang[#], Li Zhu[#], Yuan Zhong, Lixin Xu, Chunhui Lang, Jian Chen, Fei Yan, Jiawei Li, Juhui Qiu, Yidan Chen, Da Sun, Guixue Wang, Kai Qu[*], Xian Qin[*], Wei Wu[*]

Dr. K. Zhang, L. Zhu, Y. Zhong, Prof. J. Qiu, Y. Chen, Prof. G. Wang, Dr. K. Qu, Dr. X. Qin, Prof. W. Wu

Key Laboratory for Biorheological Science and Technology of Ministry of Education, State and Local Joint Engineering Laboratory for Vascular Implants, Bioengineering College of Chongqing University

Chongqing, 400030, China

E-mail: qukaigood@cqu.edu.cn (K. Qu); qinxian224@cqu.edu.cn (X. Qin); david2015@cqu.edu.cn (W. Wu)

Dr. K. Zhang, L. Xu, Dr. C. Lang, Dr. J. Chen, Dr. F. Yan, Dr. J. Li, Dr. K. Qu, Dr. X. Qin

Chongqing University Three Gorges Hospital, Chongqing Municipality Clinical Research Center for Geriatric diseases

Chongqing, 404000, China

Prof. G. Wang, Prof. W. Wu

Jin Feng Laboratory

Chongqing, 401329, China

Dr. D. Sun

Institute of Life Sciences & Biomedical Collaborative Innovation Center of Zhejiang Province, Wenzhou University

Wenzhou, Zhejiang 325035, China

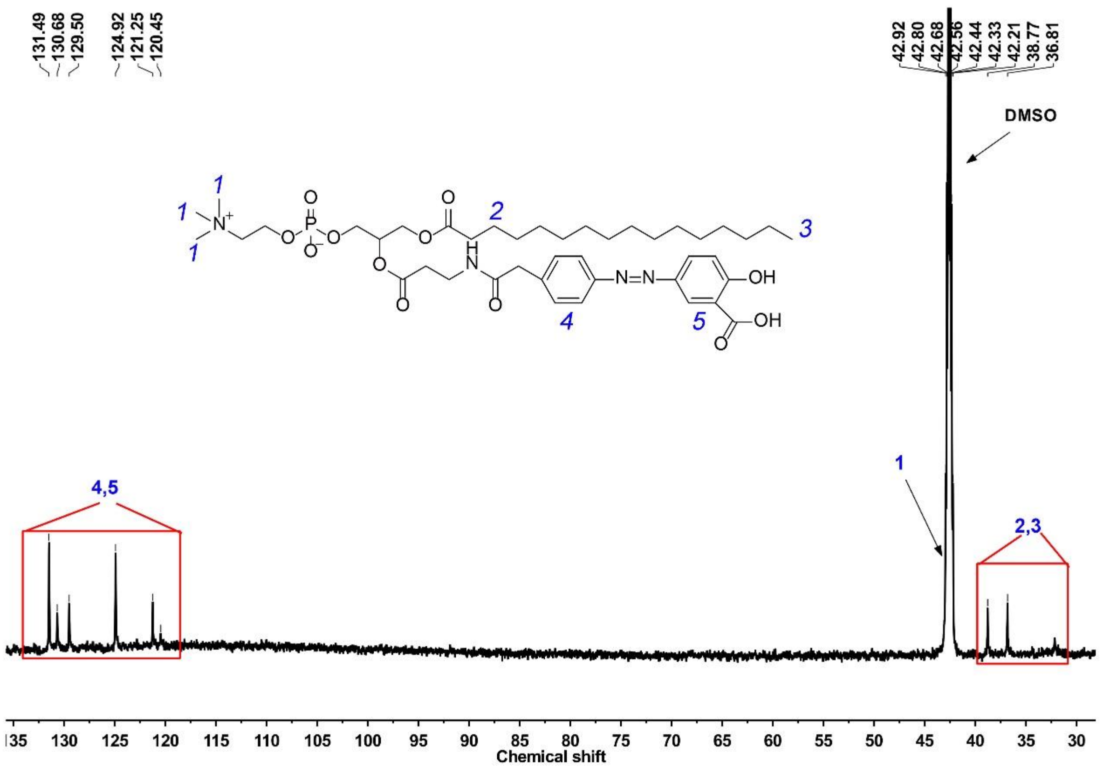

Figure S1. ^{13}C nuclear magnetic resonance spectroscopy (^{13}C NMR) of LPC-Bal.

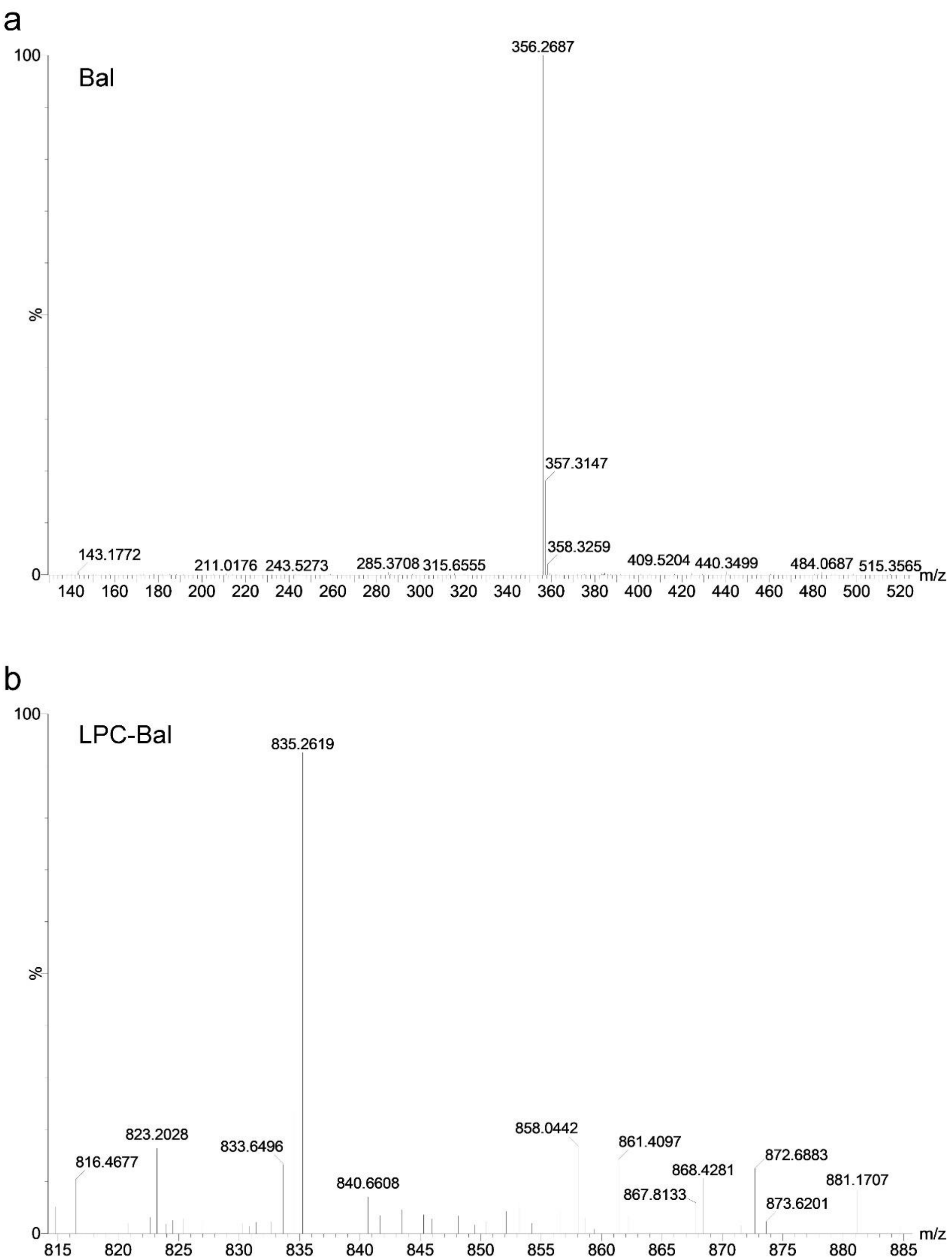

Figure S2. Mass spectrum (MS) of a) Bal and b) LPC-Bal.

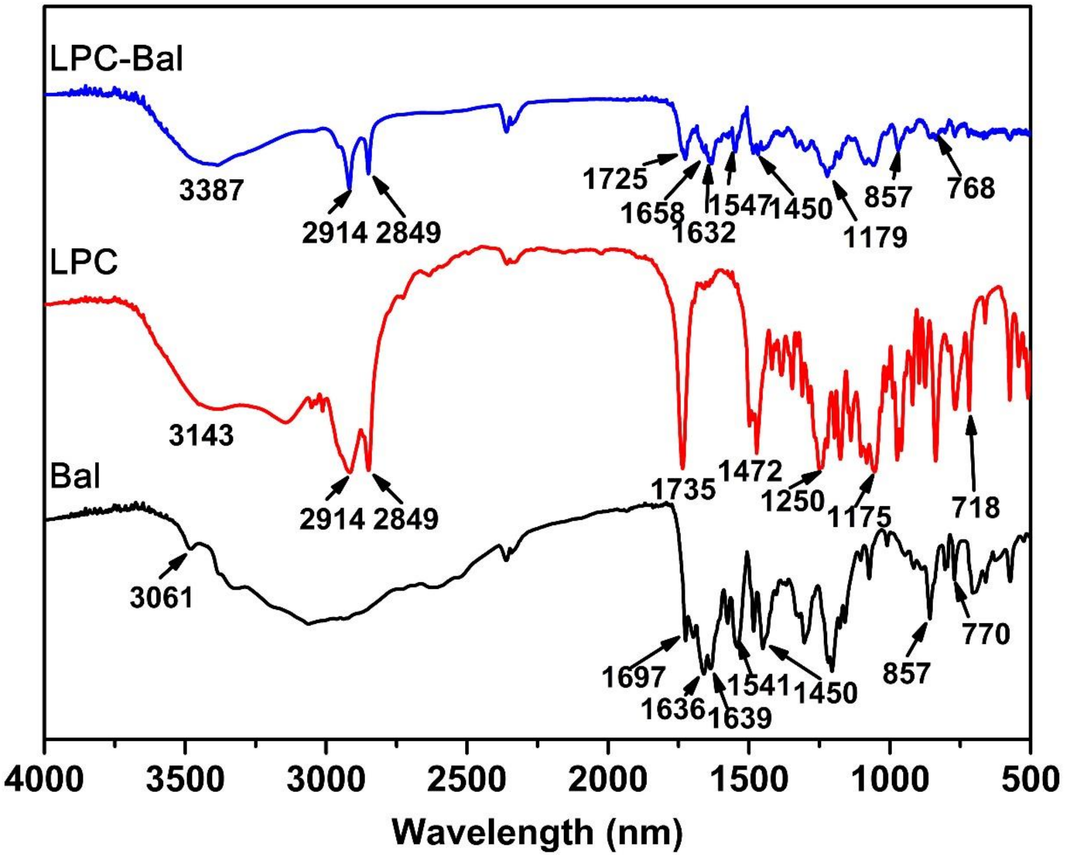

Figure S3. FT-IR spectra of free Bal, LPC, and LPC-Bal.

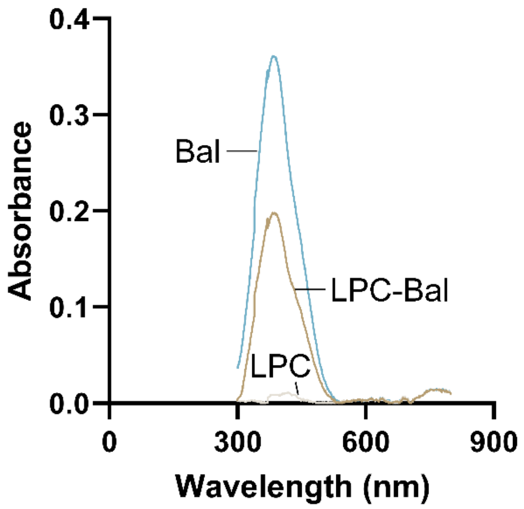

Figure S4. Characterization of LPC-Bal. UV-Vis absorption spectra of Bal, LPC, and LPC-Bal.

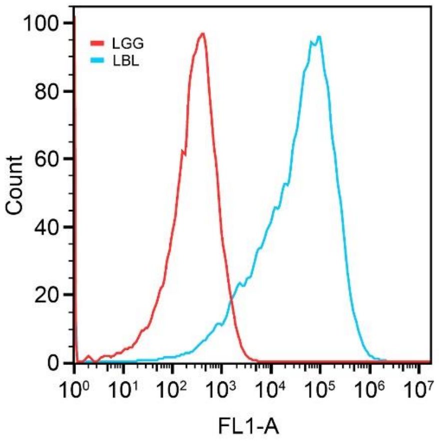

Figure S5. Flow cytometric analysis of native and decorated LGG. The coating was stained with FITC-DSPE-mPEG$_{2000}$.

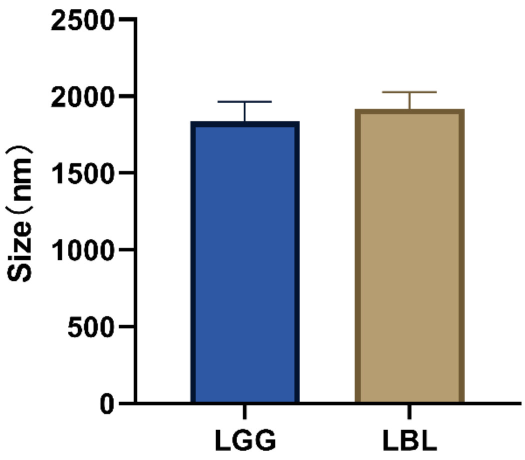

Figure S6. Characterization of LBL. Particle size distribution of uncoated LGG and LBL measured by DLS (n = 3).

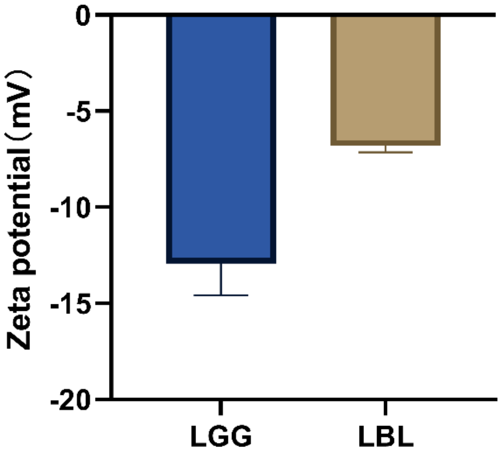

Figure S7. Characterization of LBL. ζ-potential of uncoated LGG and LBL measured by DLS (*n* = 3).

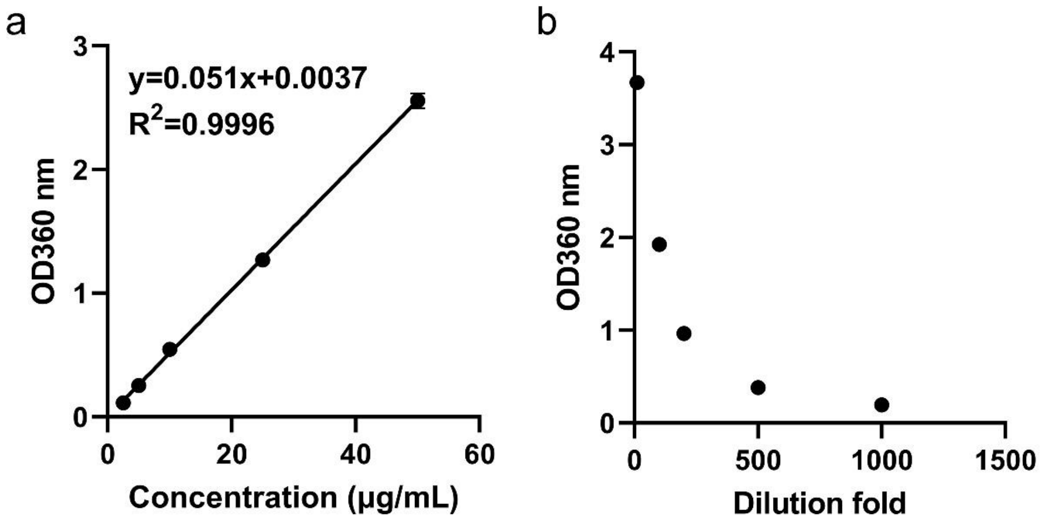

Figure S8. Calculation of Bal loading efficiency. a) Standard curve of Bal absorbance measured at 360 nm. The LBL powder was dissolved in DMSO and the absorbance of gradient dilutions at 360 nm was measured using an ultraviolet spectrophotometer. b) The relationship between the dilution fold and the absorbance.

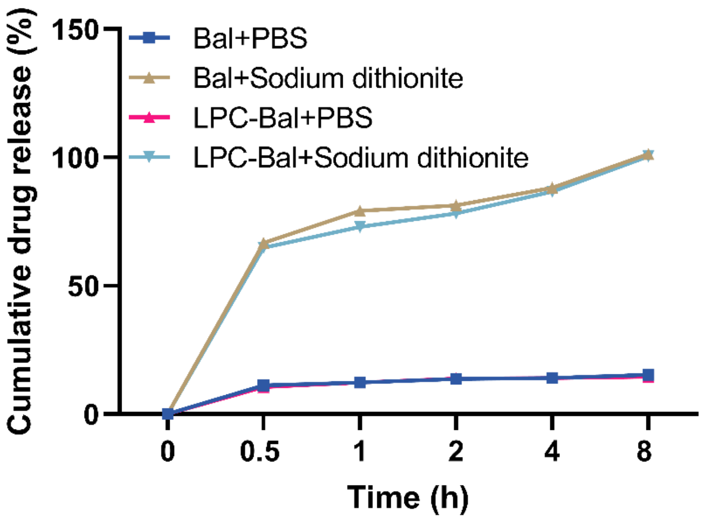

Figure S9. The cumulative release curve of Bal and LPC-Bal under SGF with or without sodium dithionite.

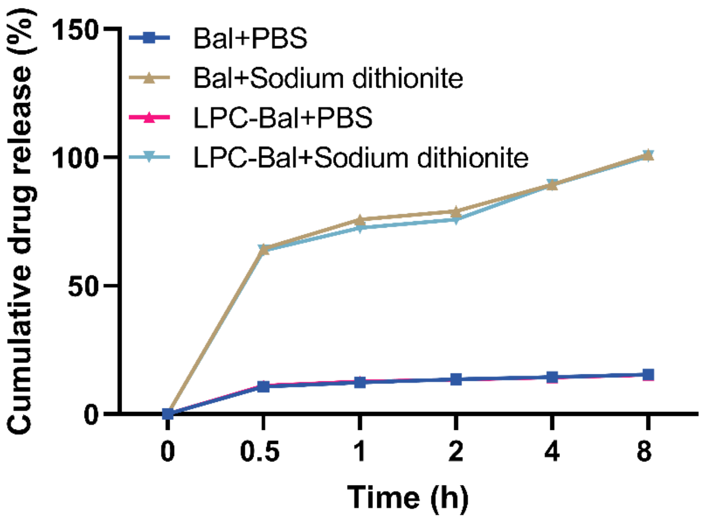

Figure S10. The cumulative release curve of Bal and LPC-Bal under SIF with or without sodium dithionite.

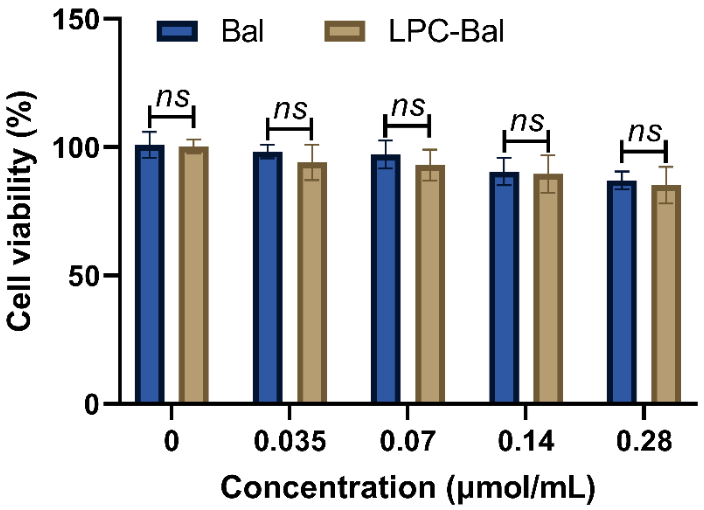

Figure S11. Cell viability of Caco-2 cell. Caco-2 cell was incubated with different concentrations of Bal or LPC-Bal and then detected with CCK-8 assay.

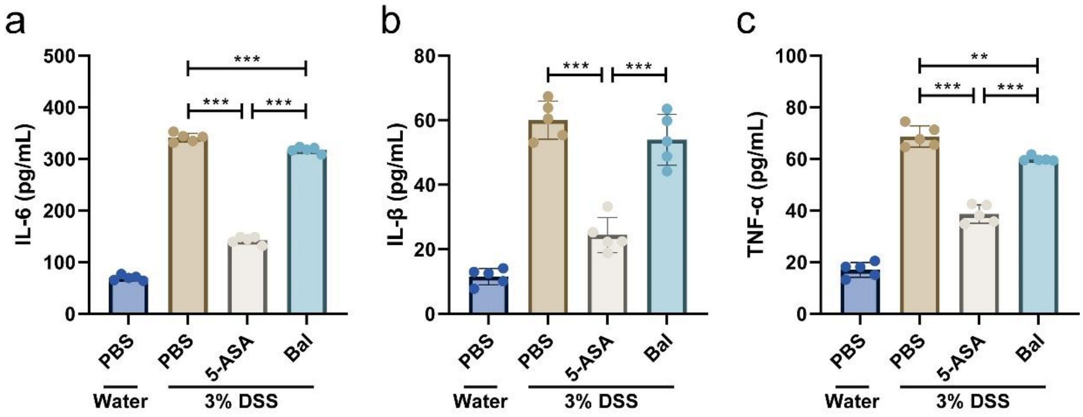

Figure S12. The contents of proinflammatory factors in DSS-induced RAW 264.7 cells. a) IL-6. b) IL-1β. c) TNF-α.

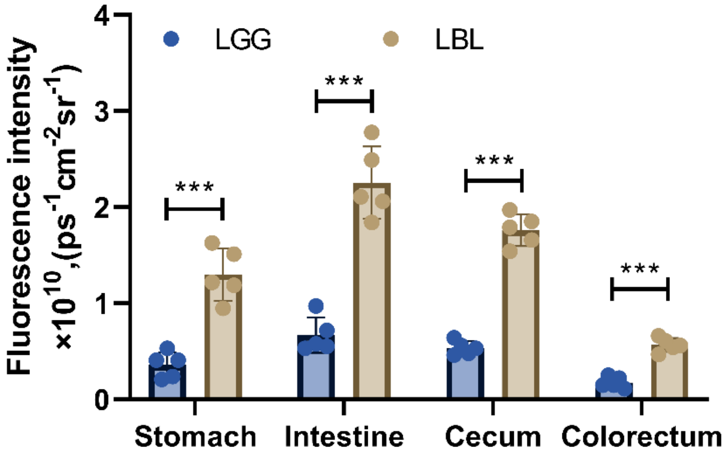

Figure S13. Statistical result of the fluorescence intensity. Fluorescence intensity of the stomach, intestine, cecum, and colorectum was calculated by IVIS. Data are shown as mean ± SD (n = 5). Unpaired t test (two-tailed) was performed, $P < 0.001$ (***).

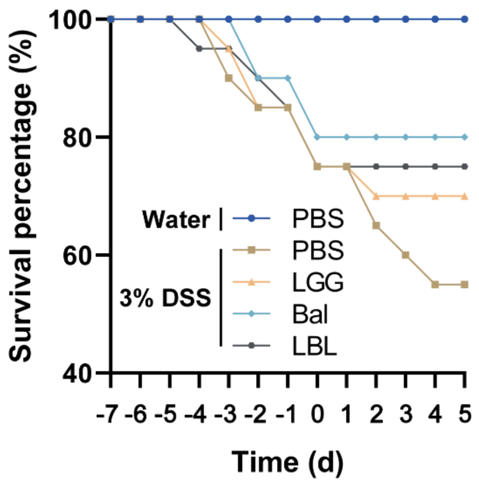

Figure S14. Survival curves of the mice. Overall Kaplan–Meier survival curves during the experimental period (*n* = 5).

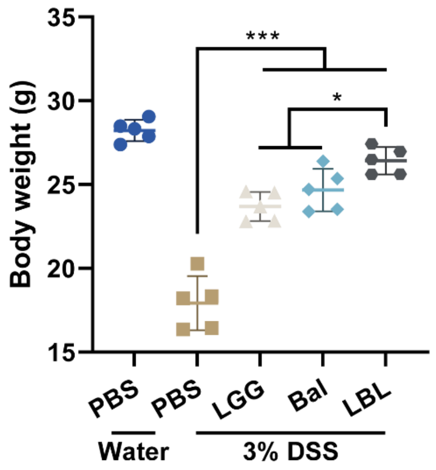

Figure S15. Body weight of the mice. Changes in mouse body weights with time. Data are shown as mean ± SD (n = 5). Significances were determined by one-way ANOVA, followed by post hoc pairwise comparisons with the Tukey honest significant difference. $P < 0.05$ (*), $P < 0.001$ (***).

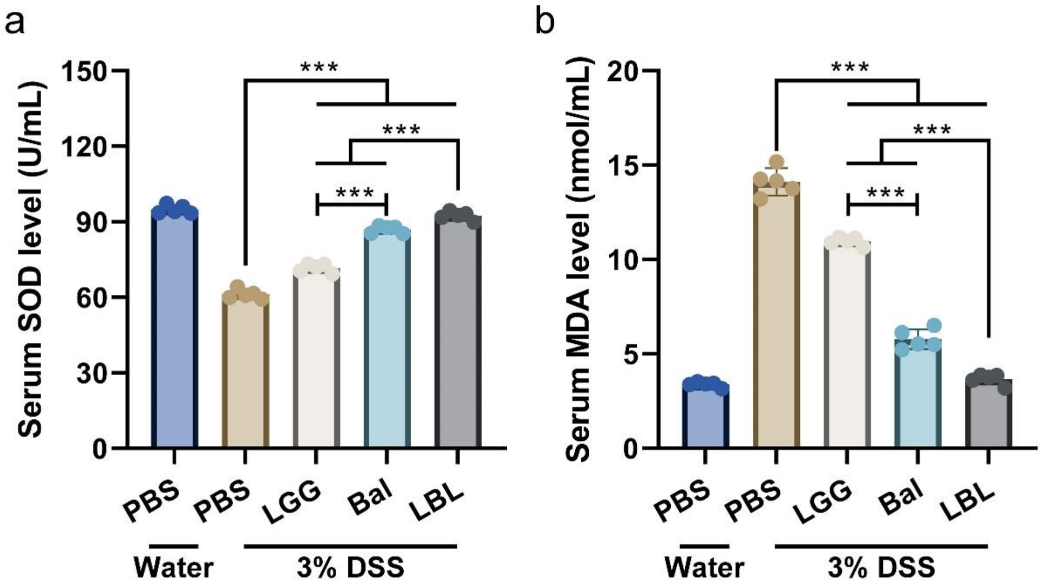

Figure S16. Suppression effect of LBL on oxidative stress. a)-b) Serum levels of SOD and MDA (n = 5). Data are shown as mean ± SD. Significances were determined by one-way ANOVA, followed by post hoc pairwise comparisons with the Tukey honest significant difference. $P <$ 0.001 (***).

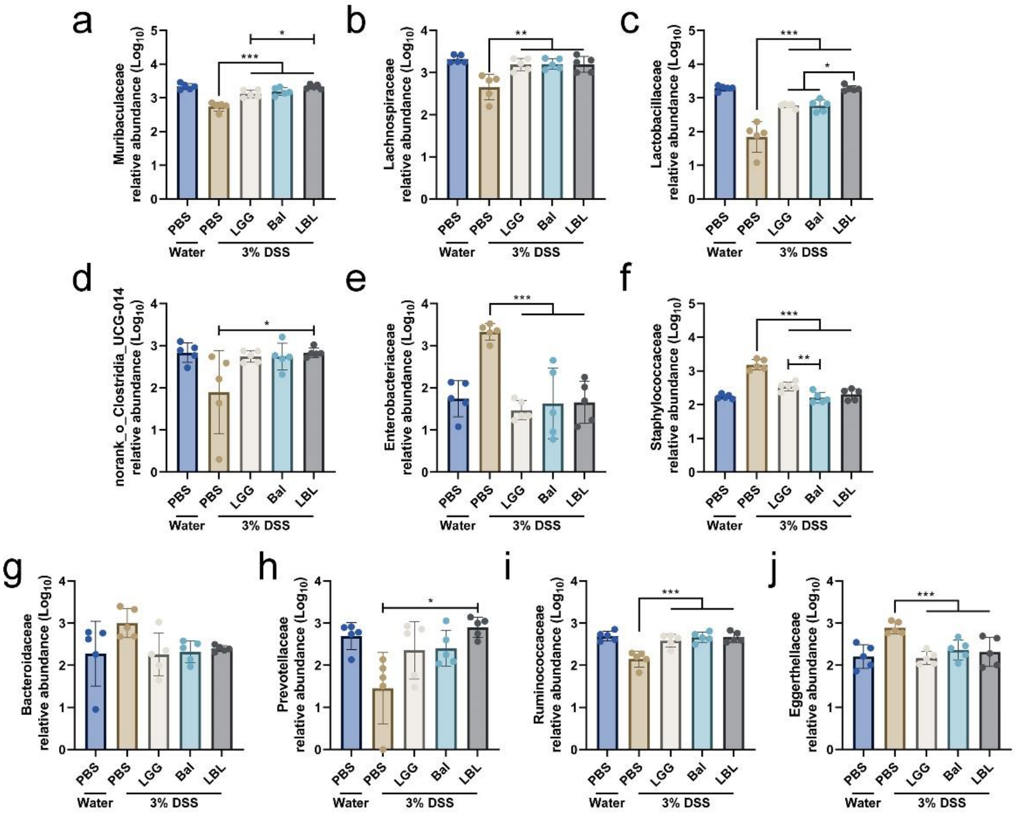

Figure S17. The statistical analysis of the 10 most abundant taxa at the family level. The relative abundance of a) *Muribaculaceae*. b) *Lachnospiraceae*. c) *Lactobacillaceae*. d) *norank_o_Clostridia_UCG-014*. e) *Enterobacteriaceae*. f) *Staphylococcaceae*. g) *Bacteroidaceae*. h) *Prevotellaceae*. i) *Ruminococcaceae*. j) *Eggerthellaceae*. Data are shown as mean ± SD (n = 5). Significances were determined by one-way ANOVA, followed by post hoc pairwise comparisons with the Tukey honest significant difference. $P < 0.05$ (*), $P < 0.001$ (***).

Table S1 The sequences of primers used in this study.

Genes	Primer sequences (5'-3')		Reference
IL-6 (mouse)	forward:	ACGGCCTTCCCTACTTCACA	This study
	reverse:	TCCAGTTTGGTAGCATCCATCAT	
IL-1β (mouse)	forward:	GAAATGCCACCTTTTGACAGTGATG	This study
	reverse:	TTCTCCACAGCCACAATGAGT	
TNF-α (mouse)	forward:	ACCCTCACACTCACAAACCA	This study
	reverse:	TAGCAAATCGGCTGACGGTG	
IL-6 (human)	forward:	CAGATTTGAGAGTAGTGAGGAAC	This study
	reverse:	GCAGAATGAGATGAGTTGTCATG	
IL-1β (human)	forward:	TACGAATCTCCGACCACCAC	This study
	reverse:	TGCAGTTCAGTGATCGTACAG	
TNF-α (human)	forward:	CTTCTCGAACCCCGAGTGAC	This study
	reverse:	ATGAGGTACAGGCCCTCTGA	
